# Editorial: Wearable sensing of movement quality after neurological disorders

**DOI:** 10.3389/fphys.2023.1156520

**Published:** 2023-02-10

**Authors:** Mohamed Irfan Mohamed Refai, Bert-Jan F. van Beijnum, Jaap H. Buurke, Peter B. Shull, Peter H. Veltink

**Affiliations:** ^1^ Biomedical Signals and Systems, University of Twente, Enschede, Netherlands; ^2^ Biomechanical Engineering, University of Twente, Enschede, Netherlands; ^3^ Roessingh Research and Development, Enschede, Netherlands; ^4^ Mechanical Engineering, Shanghai Jiao Tong University, Shanghai, China

**Keywords:** wearabe sensors, movement quality assessment, movement impairment, gait, arm use

Stroke, a leading cause of disability results in motor impairment and loss of certain functions. Recovery of movement quality takes place through a combination of spontaneous and learning-dependent processes. Eventually, motor patterns either return to more normal pre-stroke patterns (restitution) or manifest as new patterns different from those pre-stroke (compensation) (Bernhardt et al., 2017; Kwakkel et al., 2019; Vliet et al., 2020). Objective measurement of movement quality can help us understand recovery post stroke, and tailor patient specific therapies during rehabilitation.

Wearables have been used extensively to measure movement biomechanics after neurological disorders (Dobkin and Martinez, 2018). Physical and novel machine learning models applied to data collected from wearables have allowed us to extract low and high-level parameters such as activity and pose of the human body. Miniature sensors can be used by the clinicians to setup more measurements post stroke, and also help track motor recovery at the patient’s living space.

This Research Topic gathered studies that developed or validated wearable sensors for advancing movement rehabilitation post stroke. Six high quality articles highlight the state-of-the-art in the contribution of wearables at different stages of stroke rehabilitation.

The work of Werner et al. explored how wearables can be used to administer widely accepted clinical scales for movement quality assessment. The group validated the usability of inertial measurement units (IMUs) in measuring Action Research Arm Test (ARAT) using machine learning approaches. This approach enables rapid and minimally supervised application of ARAT scores in the clinic. However, the authors of this study suggest that we need to move from discrete and subjective clinical scores and use objective measures when using wearables. The editorial team agrees that this is a challenge, given that it is currently unclear what kinematic or kinetic metrics reflect movement quality post stroke (Saes et al., 2022). Addressing gait quality, Huber et al. found that assessing a parameter “Walk Ratio” using a GPS watch showed excellent test-retest agreement, reliability and concurrent validity in healthy adults and chronic stroke survivors walking at least 1 m/s. This is an important finding as it strengthens the argument for using wearables in assessing movement quality in a real-life scenario. These two studies provide avenues for commercializing wearables with targeted applications of addressing movement quality post stroke.

Analysing movement quantity and quality in a wearable and real-life setting requires good activity classification algorithms. Subash et al. showed that machine learning approaches are better than classical thresholding approaches for classifying functional movements of the upper limb. Pohl et al. validated this in an ecologic context by following the participants at their homes and using a semi-naturalistic protocol. The classifier developed by this group is available for public use. Furthermore, Pohl et al. also worked on identifying gait and posture using the ecologically valid context. These well-defined studies show the strength of wearables and machine learning algorithms in activity classification which adds to the extensive literature of activity classification.

Finally, Song et al. explored how wearables can improve therapy post stroke. The group designed and tested a gamification approach using multimodal sensing to train reaching and grasping tasks relevant to rehabilitation. They also demonstrated how the approach can stimulate motivation of the participants post stroke. The study offers potential commercialization opportunities that addresses therapy post stroke at a remote clinic or at the participant’s home.

The multi-country Research Topic has a strong research presence stemming from Switzerland, in addition to China, India, Belgium, and Australia. The studies in this Research Topic contribute towards the standardization effort that is highlighted by the Stroke Recovery and Rehabilitation Roundtable (Kwakkel et al., 2019).

Five of the six articles in this Research Topic used IMUs demonstrating its ubiquitous nature and their versatility. All studies demonstrated a higher Technology Readiness Level (NASA, 2012) and shows that the stroke rehabilitation research field is moving rapidly towards commercially available systems. We recommend governments to harness this growth to improve and standardize stroke rehabilitation.

## References

[B1] Bernhardt J. Hayward K. S. Kwakkel G. Ward N. S. Wolf S. L. Borschmann K. (2017). Agreed definitions and a shared vision for new standards in stroke recovery research: The stroke recovery and rehabilitation roundtable taskforce. Neurorehabil. Neural Repair 31, 793–799. 10.1177/1545968317732668 28934920

[B2] Dobkin B. H. Martinez C. (2018). Wearable sensors to monitor, enable feedback, and measure outcomes of activity and practice. Curr. Neurol. Neurosci. Rep. 18, 87. 10.1007/s11910-018-0896-5 30293160

[B3] Kwakkel G. Van Wegen E. Burridge J. H. Winstein C. van Dokkum L. Alt Murphy M. (2019). Standardized measurement of quality of upper limb movement after stroke: Consensus-based core recommendations from the Second Stroke Recovery and Rehabilitation Roundtable. Int. J. Stroke 14, 783–791. 10.1177/1747493019873519 31510885

[B4] NASA (2012). Technology readiness level. Available at: https://www.nasa.gov/directorates/heo/scan/engineering/technology/technology_readiness_level (Accessed 31 1, 2023).

[B5] Saes M. Mohamed Refai M. I. van Beijnum B. J. F. Bussmann J. B. J. Jansma E. P. Veltink P. H. (2022). Quantifying quality of reaching movements longitudinally post-stroke: A systematic review. Neurorehabil. Neural Repair 36, 183–207. 10.1177/15459683211062890 35100897 PMC8902693

[B6] Vliet R. Selles R. W. Andrinopoulou E. Nijland R. Ribbers G. M. Frens M. A. (2020). Predicting upper limb motor impairment recovery after stroke: A mixture model. Ann. Neurol. 87, 383–393. 10.1002/ana.25679 31925838 PMC7065018

